# Response of Soybean Root to Phosphorus Deficiency under Sucrose Feeding: Insight from Morphological and Metabolome Characterizations

**DOI:** 10.1155/2020/2148032

**Published:** 2020-08-21

**Authors:** Ahui Yang, Lingjian Kong, Hui Wang, Xingdong Yao, Futi Xie, Haiying Wang, Xue Ao

**Affiliations:** College of Agronomy, Shenyang Agricultural University, Shenyang 110866, China

## Abstract

Phosphorus (P) is one the least available essential plant macronutrients in soils that is a major constraint on plant growth. Soybean (*Glycine max* L.) production is often limited due to low P availability. The better management of P deficiency requires improvement of soybean's P use efficiency. Sugars are implicated in P starvation responses, and a complete understanding of the role of sucrose together with P in coordinating P starvation responses is missing in soybean. This study explored global metabolomic changes in previously screened low-P-tolerant (Liaodou, L13) and low-P-sensitive (Tiefeng 3, T3) soybean genotypes by liquid chromatography coupled mass spectrometry. We also studied the root morphological response to sucrose application (1%) in P-starved soybean genotypes against normal P supply. Root morphology in L13 genotype has significantly improved P starvation responses as compared to the T3 genotype. Exogenous sucrose application greatly affected root length, root volume, and root surface area in L13 genotype while low-P-sensitive genotype, i.e., T3, only responded by increasing number of lateral roots. Root : shoot ratio increased after sucrose treatment regardless of P conditions, in both genotypes. T3 showed a relatively higher number of differentially accumulated metabolites between P-starved and normal P conditions as compared to L13 genotype. Common metabolites accumulated under the influence of sucrose were 5-O-methylembelin, D-glucuronic acid, and N-acetyl-L-phenylalanine. We have discussed the possible roles of the pathways associated with these metabolites. The differentially accumulated metabolites between both genotypes under the influence of sucrose are also discussed. These results are important to further explore the role of sucrose in the observed pathways. Especially, our results are relevant to formulate strategies for improving P efficiency of soybean genotypes with different P efficiencies.

## 1. Introduction

A wide range of mineral nutrients is required by plants to carry out vital processes for normal growth and development. Phosphorous (P) is the second most limiting macronutrient in crop production after N and is an important component of nucleic acids, proteins, lipids, sugars, energy molecules (ATP and ADP), and NADPH, implying that P has a central role in molecular structures of major cellular molecules and is indispensable for plant cell functioning [1, 2]. The focus in plant breeding has been to improve crop production and reduce plant's reliance on inorganic P fertilizers. The increasing knowledge on the sensing and responses to P starvation in plants is increasing but much still remains undiscovered. A complex series of signaling cascades has been suggested which control P starvation responses in plants. The role of transcription factors (TFs) such as PHR1 (a MYB TF) and its interaction with P starvation-induced genes and ubiquitin-like modifier (SUMO) E3 ligase (SIZ1) has been revealed in P starvation responses [3–5]. MicroRNAs also interact with PHR1 as intermediate components of the signaling cascade. Additionally, transcriptional profiling studies to P starvation have also implicated other TFs like PTF, bHLH, and WRKYs in plant's response to P starvation [4]. Plants may respond differently to tissue P status and local variation in soil P availability. Systematic response of plants to P starvation may also involve a fine balance between auxin, ethylene, and cytokinin concentrations and shoot to root transport [6]. Most importantly, many recent studies have demonstrated the role of shoot-derived carbohydrate signals in the systemic modulation of plant P starvation responses [7].

Sugars in plants are derived from photosynthesis in leaves and act as substrates for energy metabolism and biosynthesis of carbohydrates [8]. Sucrose (Suc) as a signaling molecule in plants plays key roles in a wide array of plant developmental processes and differentiation [9, 10]. The Suc signaling is involved in carbon and nitrogen assimilation, starch synthesis, downregulation of CO_2_ fixation (in photosynthesis), carbohydrate synthesis and partitioning, synthesis of chlorophyll and nonphotosynthetic pigments, and nitrogen metabolism ([10] and references therein). As sugars are nutrients and metabolized extensively within plants under any particular developmental stage or stress, downstream metabolites can act as sensory molecules to sense sugars. Several catabolites and respiratory intermediates (e.g., acetate) have also been shown to affect gene expression in pathways related to phosphorus (P), energy, plant hormones, and enzymes which in turn affect plant growth and development [11]. While most well-studied Suc driven pathways/processes are associated with general plant metabolism and take place in multiple tissues simultaneously, Suc-specific signaling pathways also exist in plants such as in beet leaves. This Suc-specific response pathway can control carbohydrate (assimilate) partitioning by manipulating the expression of Suc symporters. The mRNA and the activity of Suc symporters were decreased in beet leaves after Suc treatment while other symporters (which were nonfunctional) from potato leaves (*StSUT2*; sensors) showed an increased expression [12, 13]. Further studies have demonstrated that Suc transporters may also interact with cytochrome b5 to mediate cross-membrane sugar transport into the cells and the sugar availability serves as a signal to regulate Suc transporters [14]. Whatever is the case, it is now well established that the role of Suc as a signal molecule is of utmost importance to plant life.

Many plant species have demonstrated an increase of shoot-derived carbohydrates when P availability to plant is limited [15]. Upregulation of Suc transport proteins that deliver Suc to phloem has been reported when leaf Suc concentration was increased resulting in the transport of Suc to roots [16]. Under low P conditions, the metabolism is rerouted and severe P starvation results in the production of intracellular phosphatases and nucleases, which mobilize P from cellular metabolites and nucleic acids. An increased Suc supply to roots increases the root-shoot biomass ratio and modifies root biochemistry and morphology [15]. On the contrary, when Suc translocation and/or biosynthesis are stopped, plant's response to P starvation is attenuated suggesting an important role of Suc signaling in P starvation responses. Transcriptomic response of Arabidopsis to P starvation indicated that >70% of the expressed genes can be induced by Suc [17]. Earlier reports in Arabidopsis suggested that genes related to P mobilization, uptake, signal transduction, transport, and carbohydrate metabolism, as well as enzymes of Suc metabolism, were upregulated when exogenous Suc and P were applied to leaves [7]. Increased Suc supplies to P-deprived plants influence the root growth and development as a systemic response. The transcript abundance of P-responsive genes in response to Suc application under P starvation conditions increases. However, the same happens when Suc is applied under P-replete conditions, suggesting that two signaling pathways might be involved [15, 18]. These observations in addition to the reports on the involvement of the SnRK signaling pathway in P starvation responses suggest that our knowledge about the role of Suc in P starvation signaling needs continued efforts [19].

Soybean (*Glycine max* L.) is an important source of proteins, oil, and micronutrients for humans and animals [20]. Soybean production is often limited due to low P availability in soils. It employs a wide set of morphological, physiological, and biochemical responses to P deficiency. Major works on soybean's response to P starvation have demonstrated the identification of P-responsive genes and P transporters [21–24]. Previous studies on soybean have demonstrated the presence of genotypes with different P efficiencies [25]. Such genotypes are important genetic resources for a clear understanding of transcriptional, metabolomic, morphological, and physiological response of soybean to P deficiency. A recent study adopted a combined transcriptomic and metabolomic approach and demonstrated that complex molecular responses scavenging internal P from phosphorylated metabolites are adopted by P-efficient genotypes [26]. While previous studies enhanced our understanding towards P deficiency-driven responses in soybean, no report has demonstrated the role of sugars in soybean's P starvation responses. With the increasing knowledge on P- and sugar-driven responses to P starvation, it is important to elucidate changes within soybean roots in this regard. The focus of our study was to test the biological response of soybean root system to the exogenous Suc application by using a previously identified low-P-sensitive genotype. Furthermore, we aimed to study the synergistic effects of P starvation and Suc application on root morphological traits, i.e., root length, root surface area, root volume, and number of lateral roots, and whole metabolome.

## 2. Materials and Methods

### 2.1. Plant Material and Growth Conditions

In this study, we used a low-P-tolerant and a low-P-sensitive soybean genotype, i.e., Liaodou (L13) and Tiefeng 3 (T3), respectively [27]. For experiments, five soybean seeds were first surface sterilized with H_2_O_2_ and germinated in culture bags (8 × 16 cm; diameter × length) in three replicates laid out in a split-plot experimental design. Seed germination was achieved in 48 h in the dark, and then, the bags were shifted to culture rooms under 16 h light/8 h dark cycles with the light intensity of 200 ± 20 *μ*mol m^−2^s^−1^ during light cycles at 18-28°C in a distilled water culture. Prior to shifting to culture rooms, two seedlings were removed and three uniform seedlings were retained. Once the soybean seedlings reached VE stage, the distilled water was replaced with a nutrient solution containing 4.5 mmol·L^−1^ KNO_3_, 1.2 mmol·L^−1^ NH_4_NO_3_, 3.6 mmol·L^−1^ CaSO_4_·_2_H_2_O, and 0.25 mmol·L^−1^ MgSO_4_. Trace elements were 9 *μ*mol·L^−1^ H_3_BO_3_, 0.9 *μ*mol·L^−1^ MnSO_4_, 0.9 *μ*mol·L^−1^ ZnSO_4_, 1.5 *μ*mol·L^−1^ CuSO_4_, 0.18 *μ*mol·L^−1^ (NH_4_) 6Mo_7_O_24_, and 9 *μ*mol·L^−1^ Fe-EDTA. Two Suc levels were maintained, i.e., 0% Suc (-S) and 1% Suc (+S, 0.0292 mM (with purity > 99.9%)). For P treatment, two concentrations were applied, namely, P-deficient (0 mmol·L^−1^ KH_2_PO_4_ with KCl to maintain the potassium salt concentration consistent with normal P supply, -P) and normal +P (0.5 mmol·L^−1^ KH_2_PO_4_). During the experiment, the pH value of nutrient solution was maintained at 5.8 by adding diluted HCl or KOH each day. Uniform seedlings were harvested after 9 days after Suc and P treatments. The average value of three plants per bag was taken as one repetition, and three repetitions were taken for each treatment for root morphological investigation, while for metabolomic studies, eight replicates were used. Once the whole seedlings were removed, they were washed with distilled water and the shoots and root system of the seedlings were separated from the cotyledonary nodes. Root length, root surface area, root volume, and the number of lateral roots were evaluated by the computer image analysis software (WinRHIZO root analysis system, Regent Instruments Inc. Quebec, Canada). The root and shoot weights were recorded and the root : shoot ratio was estimated.

### 2.2. Sucrose Content Determination in Root Samples

For the determination of Suc content, 0.4 mL ethanol extract was added to 200 *μ*L NaOH (2 M) and boiled at 100°C in water bath for 5 min followed by cooling at room temperature. First, 2.8 mL of 30% HCl was added and then, 0.8 mL benzene diphenol was added. The mixture was put in a water bath shaker for 10 min at 80°C, removed from water bath, and cooled at room temperature; optical density (OD) values were recorded by using a spectrophotometer (Shanghai MAPADA Instruments Inc., China) at 480 nm and compared with the standard curve; standard curve was generated by taking a series of Suc solution (0-100 *μ*g·mL^−1^) and measuring their OD.

### 2.3. Metabolite Analysis

#### 2.3.1. Global Metabolomics Analyses

Root samples were collected from each treatment in eight replicates and submitted to widely targeted metabolomics analysis. The sample preparation, extract analysis, metabolite identification, and quantification were performed at Chaya Biotechnology Co., Ltd., Shanghai (http://www.chayabiotec.com/), following their standard procedures.

#### 2.3.2. Sample Preparation

All samples were grinded to fine powder using a Grinding Mill at 65 Hz for 90 s. A total of 50 mg of sample was weighed and extracted with 800 *μ*L of methanol. The samples were vortexed for 30 s and centrifuged at 12000 rpm and 4°C for 15 min. 200 *μ*L of supernatant was transferred to a vial for LC-MS analysis.

#### 2.3.3. Liquid Chromatography Coupled Mass Spectrometry

The data acquisition instrument system included LC-MS (Thermo, Ultimate 3000LC, Orbitrap Elite). The liquid phase conditions included (1) column: Hypergod C_18_ (100 × 4.6 mm 3 *μ*m); (2) mobile phase: phase A = water + 0.1%formic acid, phase B = acetonitrile + 0.1%formic acid; (3) elution gradient: 0 min B = 5% in comparison, 2 min B = 5%; B was linearly increased to 95% in 12 min and maintained at 95% for 15 min, and B was reduced to 5% and was balanced to 17 min; and (4) flow rate 0.3 mL·min^−1^; injection volume = 4 *μ*L, and automatic injector temperature = 4°C, whereas the MS conditions were as follows: the positive electrospray ionization (ESI+) temperature was 300°C, sheath gas flow rate was 45 arb, aux gas flow rate was 15 arb, sweep gas flow rate was 1 arb, spray voltage was 3.0 KV, capillary temperature was 350°C, and S-Lens RF level was 30%. The ESI- conditions were the same as ESI+ except that the spray voltage was 3.2 KV and the S-Lens RF level was 60%.

## 3. Statistical Analysis

The morphological data analysis was done in Microsoft Excel 2013. The feature extraction of data was performed, and it was preprocessed with SIEVE software (Thermo) and then normalized and edited into two-dimensional data matrix by excel 2010 software, including retention time (RT), compound molecular weight (compMW), observations (samples), and peak intensity. Following data filtering, multivariate analysis (MVA) was done using SIMCA-P software (Umetrics AB, Umea, Sweden).

For statistical analysis, missing values were assumed to be below the limits of detection, and these values were imputed with a minimum compound value. The relative abundance of each metabolite was log transformed before analysis to meet normality. Dunnett's test was used to compare the abundance of each metabolite between the designated comparisons. False discovery rate was used for controlling multiple testing. The supervised multivariate method, partial least squares-discriminant analysis (PLS-DA), was used to maximize the metabolome difference between the comparisons. The relative importance of each metabolite to the PLS-DA model was checked using a parameter called the variable importance in projection (VIP). Metabolites with VIP > 1 and log2 fold change ≥ 1 or ≤-1 were considered differential metabolites for group discrimination. Principal component analysis, hierarchical cluster analysis, and KEGG pathway analysis were performed in R software (http://www.r-project.org).

## 4. Results and Discussion

Unlike animals, plants are sessile organisms, so they must respond to adverse conditions through adjustment in their growth and development and in metabolic activities. Through the long evolution process, plants have developed sophisticated strategies to better adapt to P starvation [28]. Phosphorus and carbon are essential elements for soybean growth and development, and unavailability of one or both of them affects the plant health [29]. Previous studies have demonstrated that plants respond to P deficiency by readjusting their growth and development and metabolic activities. The most pronounced changes are in the root architecture system, i.e., reduction of primary root growth and the formation of more lateral roots and root hairs [28]. It has been established in earlier studies that sucrose (Suc) is a global regulator of plant responses to P starvation [8, 17]. Here, we explored the metabolic and morphological changes in soybean roots with different P efficiencies.

### 4.1. Soybean Root Morphological Responses under Normal and P-Deficient Conditions with and without Sucrose Feeding

**Figure 1:**
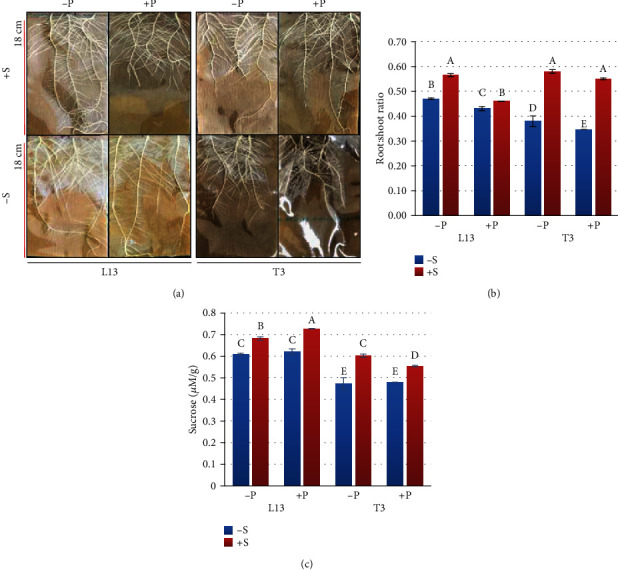
(a) Root physiological responses, (b) root : shoot ratio, and (c) root sucrose content of L13 and T3 soybean genotypes grown under P starvation (-P) and normal P (+P) levels fed with (+S) or without (-S) exogenous sucrose application. The error bars represent standard deviation, and the different letters on the bars show significantly different values.

To assess the dynamic morphological alterations of L13 (low-P-tolerant) and T3 (low-P-sensitive) roots under -P and +P conditions, we grew soybean seedling for 9 days under normal and P starvation conditions fed with or without Suc. Our results showed that Suc feeding significantly increased root length, root surface area, and number of lateral roots in L13 roots grown in P starvation conditions. Under normal P conditions, the differences were nonsignificant except for number of lateral roots between L13 roots fed with or without Suc. With exogenous Suc application, the roots were longer, with a larger surface area and volume and a higher number of lateral roots under P-deficient conditions as compared to normal P conditions in L13 (Table 1). However, under no Suc conditions, total root length and root surface area still differed significantly in L13 under P starvation conditions as compared to normal P conditions suggesting that P deficiency has a pronounced effect on root growth irrespective of Suc (Table 1). In comparison to L13, T3 was identified as a P-inefficient (low-P-sensitive) genotype [27]; hence, we checked if the morphological responses of T3 were different from L13. Suc feeding did not significantly affect the total root length, root surface area, and root volume, except number of lateral roots, during P starvation, suggesting that T3 is a P-inefficient genotype (Table 1; Figure 1(a)).

Considering these results, we can understand that Suc under P deficiency has a pronounced effect on root morphology. This further explains the fact that Suc application coordinates P deficiency-driven root morphological variations [30]. These results are clearly in agreement with a recent study by Jain et al. [31] who suggested a strong influence of Suc availability on the development of root hairs and the P-starved roots fed with Suc showed three times higher number of hairs, which is a major factor in root volume [31]. The fact that the absence of Suc still affected root length and surface area in L13 is quite relevant with the P starvation responses observed in an earlier study. The authors explained that under P starvation conditions, root hairs increase the root surface area in order to explore a greater volume of soil [32]. Therefore, the changes in root morphology in L13 in the absence of Suc are adaptations to P starvation conditions for efficient P acquisition. Similar responses have been noted in Arabidopsis, maize, and soybean [26, 30, 33, 34]. The different observations in T3 root morphological responses to P starvation responses are consistent with the previous screening results. Furthermore, it is known that low-P-insensitive genotypes respond differently than low-P-sensitive soybean genotypes as observed by Zhou et al. [25].

We further confirmed these observations by studying the root : shoot of both genotypes under the influence of exogenous Suc. Change in root : shoot ratio in plants experiencing P starvation is a well-known strategy [35]. We noted that root : shoot ratios in both L13 and T3 increased after Suc supplementation (Figure 1(b)). To check if the tested roots had correspondingly higher amounts of Suc in the treatments showing higher root : shoot ratios, we determined the level of endogenous Suc in soybean roots. The results indicated that in -S, the root Suc content in -P and +P was almost the same in L13 and a similar response was noted for T3. Suc application resulted in increased contents of endogenous Suc in both P level treated soybeans. +P showed relatively lower levels than -P in both genotypes, and L13 had higher values than T3 (Figure 1(c)). These observations indicate that Suc had pronounced effect on soybean root morphological adjustments during P starvation. Previously, it is known that plants allocate more resources to their roots in response to P status; therefore, they increase root : shoot ratios [36, 37].

Together, the root morphological data and endogenous Suc content in roots indicated that L13 has better potential to survive under -P conditions. T3 responded differently to P starvation and Suc application. Sucrose feeding significantly and positively affected L13 root responses in P-starved conditions while T3 being a low-P-sensitive genotype still showed improvements in root : shoot ratio and number of lateral roots. This is in accordance with findings of Karthikeyan et al. [30] who showed that lateral root density increased significantly in P-starved plants under sugar feeding. Furthermore, the role of root apex (lateral roots) in P sensing is previously well established in legumes [38]; therefore, we conclude that exogenous Suc application improves P sensing. Together, these observations confirm that exogenous Suc supplementation in soybean roots helps develop efficient P starvation responses.

### 4.2. Overview of the Metabolome Profiling in Soybean Roots under P-Deficient Conditions with and without Sucrose Feeding

**Figure 2:**
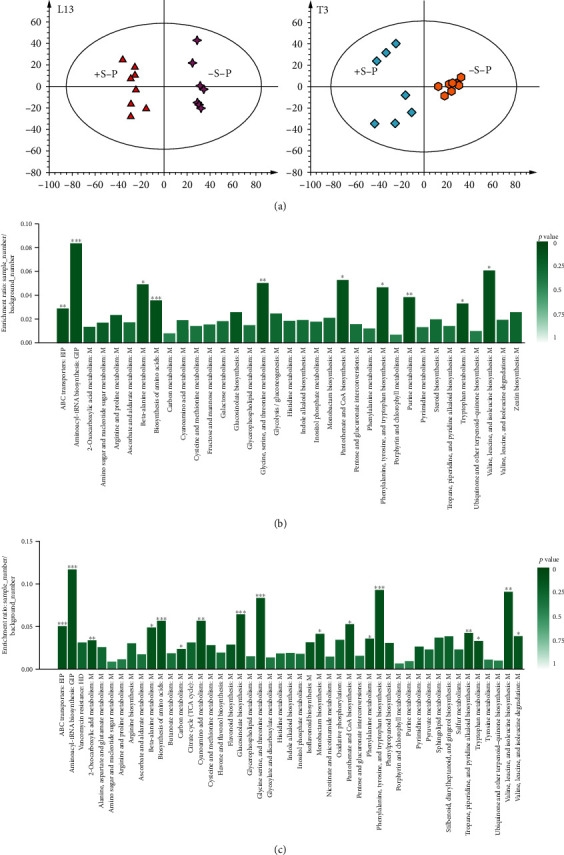
(a) Principle component analysis and KEGG pathway analysis of (b) L13 and (c) T3 soybean roots grown in P-starved conditions (-P) fed with (+S) or without sucrose (-S). ∗, ∗∗, and ∗∗∗ represent KEGG pathways significantly enriched at *p* < 0.05, 0.01, and 0.001, respectively.

Considering the role of exogenous Suc application in the root morphological response visualized in L13 and T3 soybeans, we explored the metabolite profiles of the soybean roots under P starvation conditions under the influence of Suc. We performed a principal component analysis (PCA) using metabolome data from both genotypes in the four conditions. Our data showed that the quality check (QC) samples and most of the replicates clustered together highlighting a good quality of the metabolite profiling data [39]. Moreover, we observed four groups of samples in which some were based on separation between genotypes and others were underlined by Suc and P treatments (Figure S1). Thirty differentially accumulated metabolites (DAM) were found in L13 roots under P starvation treated with or without exogenous Suc application (Supplementary Table 1). Based on DAMs, the samples were separately grouped in a principle component analysis (PCA) (Figure 2(a)). The compounds were classified into amino acids and derivatives, carbohydrates, organic compounds, indoles and derivatives, and carboxylic acids and derivatives. These metabolites were mapped through KEGG pathway analysis on 35 different pathways (Figure 2(b)). Pathway-level visualization of metabolomics data provides an essential means for capturing the systematic properties of the inner activities of tissues treated under specific conditions [40]. On the other hand, we found 46 DAMs in T3 roots under P starvation treated with or without exogenous Suc (Supplementary Table 1). The metabolites were classified as amino acids and derivatives, organic acids, carboxylic acids and derivatives, indoles and derivatives, organic acids, and others. KEGG pathway analysis showed that the DAMs were mapped on 47 different pathways (Figure 2(c)).

Previous studies on soybean genotypes screening against P starvation and normal P levels have demonstrated that P-inefficient genotypes show limited changes in root morphology index under P starvation conditions as compared to normal/high P levels [25]. On the hand, P-efficient genotypes show typical P starvation responses as noticed in our experiment. However, the information on the metabolomic changes within the P starved roots under the influence of exogenous Suc in soybean roots having different P efficiencies is yet to be explored. Below we discussed the metabolite changes within the two tested soybean genotypes' roots.

### 4.3. Effect of Exogenous Sucrose Application on Metabolome of P-Starved L13 and T3 Roots

To study the DAMs, we focused on metabolites having variable importance in projection (VIP) > 1 and log2 fold change ≥ 1 or ≤-1. These filtering criteria resulted in eight DAMs that were downaccumulated and the same number of DAMs that were upaccumulated in –S-P compared to +S-P in L13 (Table 2). The top accumulated metabolite in L13 and T3 after +S was D-glucuronic acid, and KEGG pathway enrichment showed its involvement in five different pathways, i.e., inositol phosphate metabolism, pentose and glucuronate interconversions, ascorbate and aldarate metabolism, amino sugar and nucleotide sugar metabolism, and flavone and flavonol biosynthesis suggesting a strong role in tested conditions. We also noticed the up accumulation of N-acetyl-L-phenylalanine in both genotypes under the influence of exogenous Suc. N-Acetyl-L-phenylalanine was mapped in the phenylalanine metabolism pathway. We also looked for common downaccumulated metabolites within both genotypes. Allantoin, dihydrouracil, L-nicotine, and *α*-tocotrienol were all downaccumulated in +S-treated roots as compared to -S roots frown under P-starved conditions.

**Figure 3:**
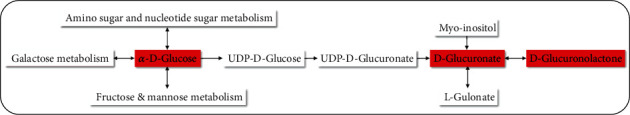
Effect of exogenous sucrose application (+S/-S) on ascorbate and aldarate metabolism in L13 and T3 soybean genotypes' roots under P starvation conditions (-P).

The increased accumulation of D-glucuronic acid in both genotypes is an important observation. It is a produced from myoinositol in inositol phosphate metabolism, which is implicated in P homeostasis in plants [41]. However, we did not find differential accumulation of myoinositol either in L13 or T3. It is important to note that UDP-D-glucose is converted into D-glucuronate in ascorbate and aldarate metabolism [42]; hence, the upaccumulation of both D-glucuronic acid and *α*-D-glucose in L13 is quite understandable. Similarly, we also found the increased accumulation of D-glucuronate and D-glucuronolactone in T3. This could be due to the exogenous Suc application as we also noticed higher Suc contents in T3 (Figure 1(c)). By comparing the DAMs between genotypes treated with -S-P, we did not find the differential accumulation of these metabolites. Hence, it is possible that both soybean genotypes responded to increased Suc accumulation by showing higher metabolites involved in ascorbate and aldarate metabolism (Figure 3). We state this because the role of Suc in differential regulation of ascorbate and aldarate metabolism is well established [43].

On the other hand, significant enrichment of the phenylalanine metabolism pathway by low-P treatment has been noted in sorghum [44]. Since Suc application affected the differential accumulation of N-acetyl-L-phenylalanine both in L13 and T3, therefore, it could be considered that phenylalanine metabolism plays a role in P starvation under the influence of Suc. The differential accumulation of allantoin is consistent with the previous reports that it accumulates in plants under the influence of stresses [45, 46]. Its downaccumulation in response to exogenous Suc applications hints that Suc has helped to relieve the P-starved soybean roots.

Finally, we compared the metabolomic response of L13 and T3 under the influence of exogenous Suc application in P-starved conditions to understand the possible mechanisms/metabolites governing better responses in L13. A relatively higher number of metabolite (110) was differentially accumulated between both genotypes (Supplementary Table 2). This is possibly due to different genetic backgrounds of the tested genotypes. It is previously known that different genotypes experiencing the same growth conditions may differ in their metabolic responses [47]. Considering our filtering criteria, we found 24 DAMs that were upaccumulated in L13 as compared to T3 (Table 3). The important upaccumulated metabolite in L13 was vanillic acid followed by inosine, ketoleucine, quercetin, suberic acid, abscisic alcohol, gibberellin A12, and Suc.

Previous studies have demonstrated the role of vanillic acid in abiotic stress tolerance in plants [48]. Vanillic acid is produced through the shikimate pathway [49]. Furthermore, in pear leaves, a simultaneous increase in Suc and vanillic acid was reported [50]. Therefore, it is possible that provision of Suc led to increase in vanillic acid, which helped L13 perform better under P starvation. More investigations on the link between vanillic acid and Suc in relation to low P response will be needed. Inosine was mapped on the purine metabolism pathway. It is known that nucleotides are composites of macronutrients (P, N, and C); therefore, possibly, the increased inosine in L13 roots is a P starvation response to increase P resources in roots experiencing P deficiency [51]. Most interestingly, the upaccumulation of gibberellin A12 is noteworthy in L13 as compared to T3. It is established that in plant roots, deficiency of sugars leads to the reduction of gibberellin synthesis. Therefore, the exogenous Suc feeding could have improved gibberellin production [52]. The accumulation of quercetin is also quite relevant to the fact that under stress, flavonoid biosynthesis is enhanced [53]. Furthermore, Suc have also been reported to regulate the enhanced induction of flavonoid biosynthesis in plants, e.g., *Morinda citrifolia* (L.) [54].

Apart from the metabolites that we screened in the filtering criteria, we noticed the regulation of the glycerophospholipid metabolism pathway. Previous studies in barley P starvation recovery have reported the regulation of this pathway [55]. In our DAMs, we found the increased accumulation of choline (log2 fold change = 0.759). The metabolite is involved in the biosynthesis of phosphatidylcholine, which is then converted into stearidonic acid through the *α*-linolenic acid metabolism pathway. A recent study had shown the downregulation of stearidonic acid under the influence of saline stress [56]. It is possible that this metabolite has similar function in P starvation as its accumulation increased in response to Suc application (which relieved plants from P stress). A further investigation could help to explore this possible assumption. Nevertheless, a more important observation was the reduced accumulation of jasmonic acid (log2 fold change = 0.796). This is consistent with previous studies where authors reported the induction of jasmonic acid pathway in P starvation [57]. Since we did not notice the DAMs associated with the glycerophospholipid metabolism or *α*-linolenic acid metabolism pathways in either of the genotypes when tested under P-starved conditions against the effect of exogenous Suc application, it could be suggested that higher choline accumulation benefited L13 by exogenous Suc application under –P conditions. In a pioneer study on the interaction between glucose-6-P and choline, it was reported that phosphocholine (which is produced from choline) is increasingly produced when Suc-starved plant cells are supplied with Suc. Therefore, our results suggest that the glycerophospholipid metabolism pathway together with *α*-linolenic acid metabolism is possibly triggered in response to increased Suc supplementation in low-P-tolerant soybean genotype.

Taken together, the metabolome analysis showed that exogenous Suc application regulates P starvation responses in soybean genotypes by possibly regulating pathways such as inositol phosphate metabolism, ascorbate and aldarate metabolism, phenylalanine metabolism, glycerophospholipid metabolism, and *α*-linolenic acid metabolism pathways.

## 5. Conclusion

This study on P-efficient and P-inefficient soybean genotypes explored the role of exogenous Suc application in P-starved roots. The root morphological evaluation confirmed that the genotypes differed in P sensitivity. The exogenous Suc application increased endogenous Suc level in root and increased lateral root number as well as root : shoot ratio in both genotypes, which are crucial strategies to adapt to P starvation. We explored differential metabolites expressed in each genotype under P starvation conditions fed with or without Suc. Metabolite profiles of both genotypes differed in their responses as numbers of metabolites were exclusively and differentially regulated within each genotype. We found three common metabolites in both genotypes, i.e., 5-O-methylembelin, D-glucuronic acid, and N-acetyl-L-phenylalanine, upaccumulated under P-starved conditions fed with Suc.

## Figures and Tables

**Table 1:** Root morphological responses of L13 and T3 soybeans grown in P-deficient (-P) and normal P (+P) conditions fed with (+S) or without sucrose (-S).

Genotype	Treatment	Total root length (cm)	Root surface area (cm^2^)	Root volume (cm^3^)	Number of lateral roots
L13	+S-P	351.48 ± 17.62^a^	57.12 ± 6.13^a^	0.74 ± 0.14^a^	408.67 ± 135.35^e^
	+S+P	287.92 ± 1.75^c^	46.53 ± 3.81^bc^	0.6 ± 0.10^abcd^	350.33 ± 68.24^de^
	-S-P	335.29 ± 10.01^b^	53 ± 4.32ab	0.67 ± 0.09^abc^	284.33 ± 9.24^bcde^
	-S+P	284.56 ± 19.45^c^	43.72 ± 4.38^cd^	0.54 ± 0.08^abcd^	273.67 ± 25.15^bcd^

T3	+S-P	204.08 ± 20.55^ef^	35.16 ± 7.24^de^	0.49 ± 0.17^cd^	270.33 ± 33.50^bcd^
	+S+P	181.94 ± 18.05^f^	35.08 ± 4.49^de^	0.54 ± 0.09^abcd^	174 ± 47.66^ab^
	-S-P	214.24 ± 20.64^def^	40.84 ± 2.22^cd^	0.62 ± 0.07^abcd^	211.33 ± 37.45^abc^
	-S+P	220.46 ± 3.32^def^	41.69 ± 3.94^cd^	0.63 ± 0.11^abcd^	217.67 ± 50.30^abcd^

Data is given as mean ± standard deviation. Different letters indicate significant different at *p* < 0.05.

**Table 2:** Differentially accumulated metabolites (VIP > 1 and log2 fold change ≥ 1 or ≤-1) accumulated in L13 and T3 soybean roots in P-starved conditions (-P) under the influence of exogenous sucrose application (+S/-S).

Compound	L13 (log2 fold change)		T3 (log2 fold change)	
	VIP	+S-P/-S-P	VIP	+S-P/-S-P
L-Phenylalanine	—	—	2.29	-1.405
3-Hydroxynorvaline	1.07	2.064	—	—
4-Hydroxybenzaldehyde	1.09	1.714	—	—
5-O-Methylembelin	—	—	1.18	1.289
Allantoin	2.44	-2.418	3.92	-1.508
Apigenin	—	—	2.00	1.501
Betaine aldehyde	4.25	1.774	—	—
Chalcone	—	—	1.07	1.060
Choline	—	—	4.81	-1.376
Coumestrol	—	—	5.99	2.053
Deoxyuridine	—	—	1.42	1.474
D-Glucuronic acid	2.70	4.812	3.41	5.475
Dihydrouracil	1.63	-1.695	2.23	-1.098
Homoserine lactone	—	—	1.05	-1.142
Indoleacetaldehyde	1.85	-7.099	—	—
Inosine	2.64	1.080	—	—
L-Nicotine	1.06	-1.590	1.49	-1.136
L-Phenylalanine	—	—	6.72	-1.398
L-Proline	1.43	1.222	—	—
L-Serine	—	—	1.01	-1.396
N-Acetyl-L-phenylalanine	1.60	1.409	2.17	2.014
p-Coumaroyl quinic acid	—	—	1.04	1.357
Pelargonidin 3-rhamnoside	—	—	1.92	1.131
Phosphocholine	1.39	-1.012	—	—
Proline betaine	3.87	-3.344	—	—
Pyrrolidine	—	—	1.19	-1.048
Rhein	—	—	1.30	1.869
S-Adenosylmethioninamine	1.95	-1.996	—	—
*α*-D-Glucose	1.51	1.104	—	—
*α*-Tocotrienol	1.02	-2.187	1.17	-1.302
*γ*-Aminobutyic acid	—	—	1.61	-1.242

**Table 3:** Differentially accumulated metabolites (VIP > 1 and log2 fold change ≥ 1 or ≤-1) accumulated between L13 and T3 soybean roots in P-starved conditions fed with sucrose.

Compound	VIP	(log2 fold change) L13/T3
Vanillic acid	1.835	4.595318
Inosine	1.847	4.317485
Ketoleucine	1.229	3.434704
Quercetin	1.507	2.949067
Suberic acid	1.633	2.876494
Abscisic alcohol	1.024	2.788464
Valerenic acid	1.150	2.526996
3-Hydroxynorvaline	1.720	2.432042
PG (22 : 2 (13Z, 16Z)/0 : 0)	1.138	2.38326
Gibberellin A12	1.641	2.344923
Pimelic acid	1.048	2.292771
CPA (18 : 1 (11Z)/0 : 0)	1.024	2.136835
Guanosine	1.008	1.854191
Phytosphingosine	1.475	1.850928
Tetrahydrofuran	1.537	1.50816
Sucrose	1.354	1.424567
LysoPE (0 : 0/16 : 0)	1.629	1.418769
S-Methylmethionine	1.062	1.290398
5-O-Methylembelin	1.725	1.139918
Chitotriose	1.038	1.122532
L-Proline	1.787	1.109779
Ornithine	1.372	1.071687
1-Monopalmitin	1.610	1.050144
Niacinamide	1.555	1.000754

## Data Availability

All the data used to support the findings of this study are included within the article and the supplementary information file(s).

## Supplementary Materials

Supplementary materials Supplementary Table 1: differentially accumulated metabolites in L13 and T3 soybean genotypes grown in P-deficient conditions with or without sucrose. Supplementary Table 2: differentially accumulated metabolites between L13 and T3 soybean genotypes grown in P-deficient conditions with sucrose. Supplementary Figure 1: principal component analysis based on metabolome data from L13 and T3 under P starvation (-P) and normal P (+P) levels fed with (+S) or without (-S) exogenous sucrose application.
